# Revealing the Interface Structure and Bonding Mechanism of Coupling Agent Treated WPC

**DOI:** 10.3390/polym10030266

**Published:** 2018-03-05

**Authors:** Jiuping Rao, Yonghui Zhou, Mizi Fan

**Affiliations:** 1 College of Material Engineering, Fujian Agriculture and Forestry University, Fuzhou 350002, China; fafurjp@163.com; 2 Department of Civil and Environmental Engineering, College of Engineering, Design and Physical Sciences, Brunel University London, Uxbridge UB8 3PH, UK

**Keywords:** wood plastic composites, interfacial optimisation, chemical reaction, adhesion, bonding mechanism

## Abstract

This paper presents the interfacial optimisation of wood plastic composites (WPC) based on recycled wood flour and polyethylene by employing maleated and silane coupling agents. The effect of the incorporation of the coupling agents on the variation of chemical structure of the composites were investigated by Attenuated total reflectance-Fourier Transform Infrared spectroscopy (ATR-FTIR) and Solid state ^13^C Nuclear Magnetic Resonance spectroscopy (NMR) analyses. The results revealed the chemical reactions that occurred between the coupling agents and raw materials, which thus contributed to the enhancement of compatibility and interfacial adhesion between the constituents of WPC. NMR results also indicated that there existed the transformation of crystalline cellulose to an amorphous state during the coupling agent treatments, reflecting the inferior resonance of crystalline carbohydrates. Fluorescence Microscope (FM) and Scanning Electron Microscope (SEM) analyses showed the improvements of wood particle dispersion and wettability, compatibility of the constituents, and resin penetration, and impregnation of the composites after the coupling agent treatments. The optimised interface of the composites was attributed to interdiffusion, electrostatic adhesion, chemical reactions, and mechanical interlocking bonding mechanisms.

## 1. Introduction

Wood plastic composites (WPC) have been considered as one of the most advanced materials, consistently growing in the last decade for uses in many industrial sectors, such as decking, automotive, siding, fencing, and outdoor furniture, mainly because of the advantages that wood material possesses, namely ubiquitous availability at low cost, biorenewability and biodegradability, low density, nontoxicity, flexibility during processing, and acceptable specific strength properties [1,2]. However, inherently highly polar and hydrophilic nature of wood flour or fibre makes it incompatible with hydrophobic and non-polar matrices, especially hydrocarbon matrices (e.g., polyethylene (PE) and polypropylene (PP)) [3,4], this may cause a problem in the composite processing and material performance, such as uneven distribution of the filler in the matrix and insufficient wetting of wood by the matrix, which results in weak interfacial adhesion and strength [2,5].

The interface of WPC is a heterogeneous transition zone extending from nanometers to microns with different morphological features, chemical compositions and mechanical properties [6,7,8]. Interfacial adhesion plays a fundamental role in the global performance of a composite. In order to formulate a reasonable WPC with optimum interface bonding, various modifications, including both physical (e.g., corona, plasma, gamma radiation) and chemical approaches (e.g., alkaline, acetylation, benzoylation, peroxide, silane, and maleated coupling agents treatments) have been attempted to decrease the hydrophilicity of wood flour, enhance the wettability of wood by matrix polymer, and eventually promote the interfacial adhesion of the constituents within the composite. With respect to the commercial production of WPC, incorporating coupling agents is probably the best available and feasible strategy for its interface optimisation [9]. 

An extensive study on the silane crosslinking of WPC and its effect on composite properties has showed that silane crosslinking can improve the adhesion between the wood filler and PE matrix by forming a set of chemical links including Si–O–C bridges, hydrogen bonds, and C–C crosslinks [10,11,12]. As a result, the strength, toughness and creep resistance of the crosslinked composite were significantly increased. Maleated olefins, such as maleated polypropylene (MAPP) or maleated polyethylene (MAPE), had been commonly reported to enhance the compatibility and interfacial adhesion of WPC by reacting with the surface hydroxyl groups of wood through the anhydride groups of the copolymers, and at the meantime entangling with the polymer matrix through the other end of the copolymers because of their similar polarities [13,14,15]. The effectiveness of these coupling agent treatments was, in general, evaluated by the improvements in the physical and mechanical properties of the composites. Nevertheless, very few studies had paid specific attention to investigate the correlation of the chemical functionalities and reactions resulted from the coupling agent treatments with other bonding scenarios (i.e., physical and mechanical bonding), and their contribution to the bonding mechanism of the composites. 

In this work, WPC materials were fabricated by the use of recycled wood flour and PE aiming at reducing the consumption of virgin raw materials and the environmental impact. The focus of this work was to optimise the interface of WPC by incorporating three different coupling agents, i.e., MAPE, bis(triethoxysilylpropyl)tetrasulfide (Si69) and vinyltrimethoxysilane (VTMS); hence, to comprehensively reveal the interface structure and bonding scenarios, and unveil the chemical, physical, and mechanical bonding mechanisms of the formulated WPC by carrying out a set of assessments including Attenuated total reflectance-Fourier Transform Infrared spectroscopy (ATR-FTIR) analysis, solid state ^13^C Nuclear Magnetic Resonance spectroscopy (NMR) analysis, Scanning Electron Microscope (SEM), and Fluorescence Microscope (FM) analyses.

## 2. Materials and Methods

### 2.1. Materials

Recycled wood flour used in this work was supplied by Rettenmeier Holding AG (Wilburgstetten, Germany), with a bulk density of 0.285 kg/m^3^, it was oven-dried at 105 °C for 24 h to remove the moisture or water before use; recycled polyethylene (PE) pellet with a bulk density of 0.96 kg/m^3^ and melt flow index (MFI) of 0.6 g/10 min at 190 °C was obtained from JFC Plastics Ltd. (Runcorn, UK); lubricants 12-HSA (12-Hydroxyoctadecanoic acid) and Struktol TPW 709 were purchased from Safic Alcan UK Ltd. (Warrington, UK); coupling agents, MAPE (500 cP viscosity at 140 °C, 0.5 wt % of maleic anhydride), Si69 (>95% purity, 250 °C boiling point) and VTMS (>98% purity, 123 °C boiling point), were purchased from Sigma-Aldrich (Dorset, UK). All of the raw materials and additives were stored in a cool and dry place before uses. 

### 2.2. Formulation of Composites

The formulation of untreated and treated WPC with specific ratios was summarised in Table 1. All of the composites were carefully prepared under the same processing condition as follows: the required amount of PE for each batch was first placed in a Brabender Plastograph twin-screw mixer and allowed to melt at 100 rpm and 190 °C for 2 min, and subsequently mixed with wood flour for 3 min. The lubricants and/or coupling agents were thus added into system and mixed for another 10 min. The resulted mixture was thus ground to pellets by using a Retsch cutting mill (SM 100, Haan, Germany). The ground blends were compression moulded on an electrically heated hydraulic press. Hot-press procedures involved 20 min preheating at 190 °C with no load applied, followed by 10 min compressing at the same temperature under the pressure of 9.81 MPa, and subsequently air cooling under load until the mould reached 40 °C. 

### 2.3. ATR-FTIR Analysis

The Fourier Transform Infrared spectroscopy (FTIR) spectra of the composites were recorded on a PerkinElmer Spectrum one Spectrometer (PerkinElmer, Shelton, CT, USA) equipped with diamond crystal and an incident angle of 45° was used. The atmospheric compensation function that minimises effect of atmospheric water and CO_2_ on the sample spectra, without the need for reference or calibration spectra. The Absolute Virtual Instrument (AVI) in PerkinElmer actively standarises instrument response to improve repeatability and protect data integrity. The instrument was operated under the following conditions: 4000–650 cm^−1^ wave number range, 4 cm^−1^ resolution, and 16 scans. The specimen dimension was 2 mm × 2 mm × 1 mm for both untreated and coupling agent treated composites, and the average of three measurements was used. 

### 2.4. Solid State ^13^C NMR Analysis

Solid state ^13^C NMR analysis was conducted on a Bruker spectrometer (Bruker BioSpin, Billerica, MA, USA) with a Cross Polarization Magic Angle Spinning (CP-MAS) probe operating at 100 MHz. The measurements were performed at ambient probe temperature with high power decoupling. Samples were packed in zirconium oxide rotors of 7 mm diameter fitted with Kel-F caps. Spectra were acquired at the spinning rate of 6 kHz, with 4096 scans per spectrum collecting in the region between −130 ppm and 270 ppm.

### 2.5. SEM and FM Analyses

All of the composites were transversely cut by using a sliding microtome with the nominal thickness of around 25 microns for the morphological investigation of the cross sections. The SEM observation was conducted on a Leo 1430VP SEM spectrometer (Carl Zeiss AG, Oberkochen, Germany) operating at 10 kV, all of the samples were conductively plated with gold by sputtering for 45 s before imaging. FM examination was conducted on a Carl Ziess Axioimager microscrope spectrometer (Carl Zeiss AG, Oberkochen, Germany) with a 100 W mercury burner, also, a green exciter-barrier filter set with 480/40 nm excitation wavelength and 510 nm emission wavelength was applied to observe the cross sections. 

## 3. Results and Discussion

### 3.1. Chemical Structure and Bonding

#### 3.1.1. FTIR Analysis

**Figure 1:**
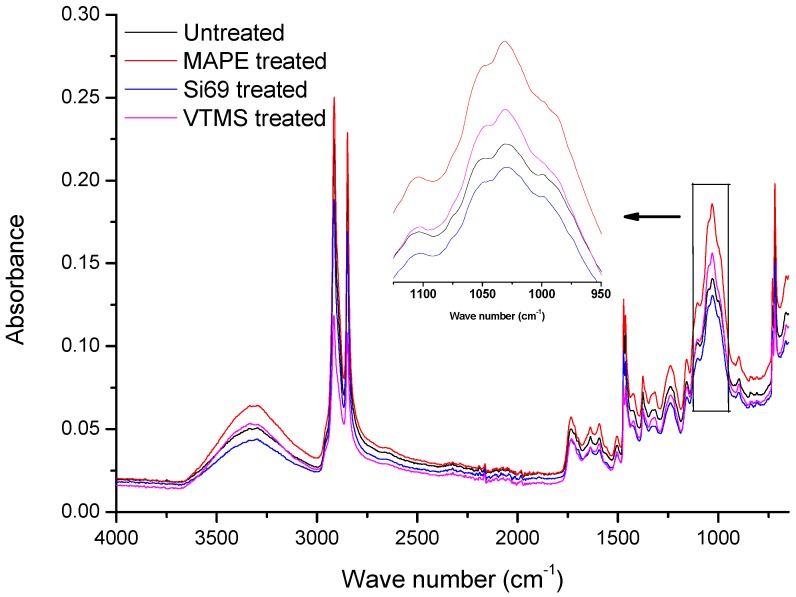
Fourier Transform Infrared spectroscopy (FTIR) spectra of untreated, maleated polyethylene (MAPE), bis(triethoxysilylpropyl)tetrasulfide (Si69), and vinyltrimethoxysilane (VTMS) treated wood plastic composites (WPC).

**Figure 2:**
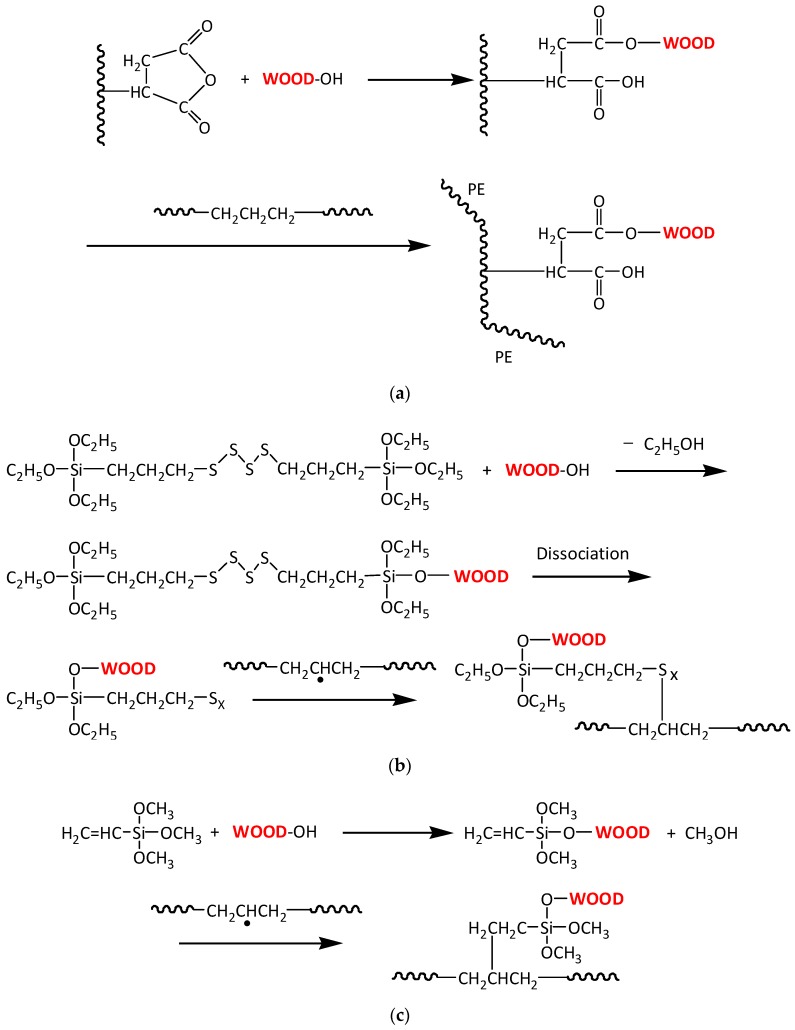
Proposed chemical reactions between the coupling agents ((**a**) MAPE; (**b**) Si69; (**c**) VTMS) and the raw materials of the composites.

Figure 1 shows the FTIR spectra of the untreated and coupling agents treated WPC. The diagnostic feature in the spectrum of MAPE treated WPC was the occurrence of more intense bands at 1637 cm^−1^ and 1734 cm^−1^, corresponding to C=C and C=O stretching vibrations [16,17], which confirmed the introduction of C=C groups and formation of ester linkages (covalent bonding) between wood particle and maleic anhydride (MA) moiety, as the reaction shown in Figure 2a. Carlborn et al. [18] reported that the regions of interest in the FTIR spectra of maleated polyolefins modified wood particles were the absorbance bands near 2900 cm^−1^ (CH stretching) and 1740 cm^−1^ (C=O stretching), suggesting the formation of ester linkages. A grafting index (GI) could be calculated by using the integrated areas under these peaks with the following equation:

(1) $$GI_{x}=\frac{A_{x\left(treated\right)}}{A_{x\left(untreated\right)}}$$

where, *x* represents the absorbance band at either 2900 cm^−1^ or 1740 cm^−1^, *A_x_* represents the integrated peak area. Accordingly, the bands of interest in the spectra of untreated and MAPE treated WPC were observed at 2915 cm^−1^ and 1734 cm^−1^, and the calculated *GI_x_* were shown as follows: *GI_2915_* = 1.14 and *GI_1734_* = 1.09. With regards to the reported MAPE modified wood particles, the corresponding *GI_2900_* and *GI_1740_* at 5% MAPE were around 1.10 and 1.25, respectively [18]. The slightly higher *GI_1740_* than *GI_1734_* resulted from the higher concentration of MAPE in the wood particles (5%) than that in WPC (3%). The spectral bands at 1031 cm^−1^ in both the spectra of untreated and MAPE treated WPC were assigned to C–O deformation and C–O–C stretching vibrations of the ethers [19]. The significant increase of the band intensity after the MAPE treatment should be resulted from the introduction of MA groups and the C–O–C covalent bonds formed between MA and wood particles.

The spectra of untreated and Si69 treated WPC did not show evident difference in terms of the band appearances and intensities, especially the bands corresponding to C–O–Si and Si–O–Si bonds (1020 cm^−1^–1100 cm^−1^), which might be an indication of very limited crosslinking reaction occurred between the coupling agent and raw materials. The disappearance of the feeble peak at 1715 cm^−1^ (C=O stretching vibration of carboxyl) and the slight shift of OH stretching vibration (shift from 3302 cm^−1^ to 3299 cm^−1^) in the spectrum of treated WPC might be resulted from the hydrogen bonding formation between wood and the hydrolysed silane (silanol). Further scrutinising of the crosslinking between Si69 and the raw materials by another analytical technique (i.e., NMR) should be of great significance for confirming the above assumptions and was carried out in the next section. 

VTMS was another coupling agent applied for refining the interface of WPC. The most distinguishing characteristic presented in the spectrum of VTMS treated WPC was the strengthened intensity of the band at 1031 cm^−1^, which was resulted from the introduced Si–O–C groups in VTMS and the Si–O–C linkages that formed between wood flour and VTMS. More importantly, it might also be attributed to the formation of Si–O–Si bonds within VTMS through the hydrolysis of the methyl ether linkages and consequent condensation with adjacent silanol groups [20,21]. When compared to that of untreated WPC, the slight reduction of intensity for the band at 1101 cm^−1^ of VTMS treated WPC might be attributed to the multiple linkages of Si–O_*n*_–Si [21]. The chemical reactions occurred between VTMS and the raw materials were proposed in Figure 2c. VTMS was firstly reacted with hydroxyl groups in wood flour by creating covalent bonds (Wood–O–Si), the hydrophobic part of the silane on the wood surface were thus chemically bonded and/or interacted through Van der Waals force with PE molecules.

#### 3.1.2. NMR Analysis

**Figure 3:**
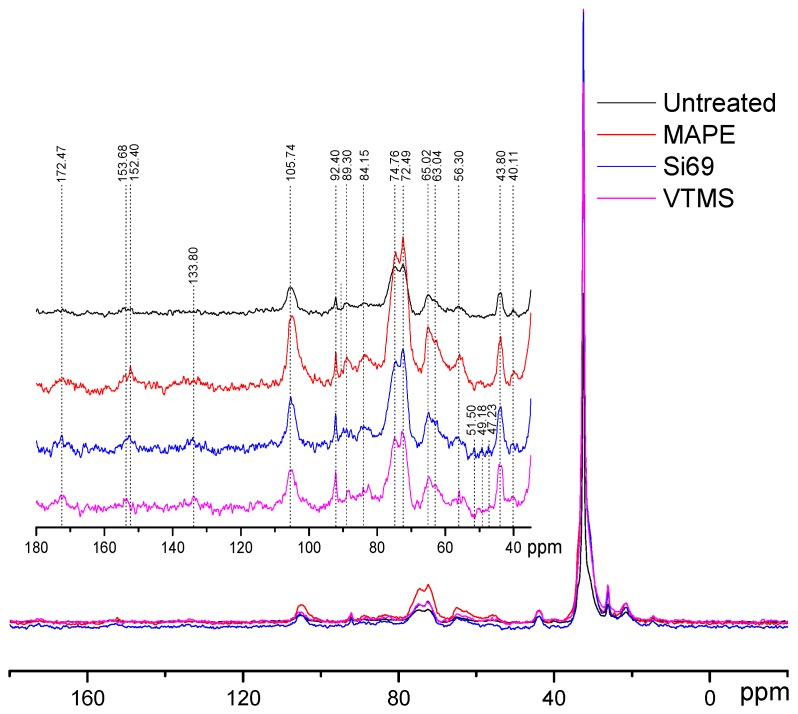
^13^C NMR spectra of untreated, MAPE, Si69 and VTMS treated WPC.

**Figure 4:**
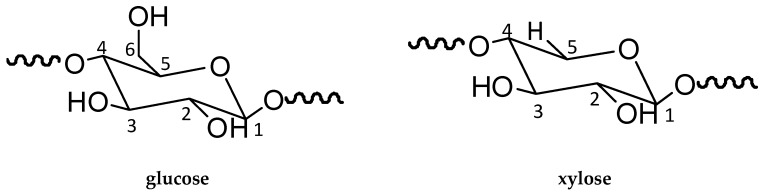
Chemical structure of cellulose unit (**glucose**) and hemicellulose unit (**xylose**).

NMR was employed to further study the effect of the incorporation of the coupling agents on the variation of chemical structure of the composites. Figure 3 shows the comparison of the NMR spectra of untreated and treated WPC. The wood component in the composites was characterised by the spectral signals of cellulose (Figure 4) at 105.74 ppm for C1, 89.30 ppm, and 84.15 ppm for C4 of crystalline and amorphous cellulose respectively, 74.76 ppm and 72.49 ppm for C2,3,5, 65.02 ppm and 63.04 ppm for C6 of crystalline and amorphous cellulose respectively [22,23,24,25,26,27]. The diagnostic signals of hemicellulose should be at around 105 ppm (C1), 84 ppm (C4), 72–75 ppm (C2,3,5), and 65 ppm (C5), which had all overlapped with the more intense signals of cellulose due to their chemical similarities [23,26]. In terms of the characteristics of lignin in wood flour, the peak observed at 172.47 ppm was assigned to carboxyl groups in lignin, peaks at 150–154 ppm and 138–132 ppm were attributed to aryl groups, and the signal at 56.3 was ascribed to methoxyl groups [22,23,26]. The resonance peaks attributed to the PE component in the composites were distinguished at the chemical shifts of 43.80 ppm and 32.51 ppm assigning to methylene groups (–CH_2_–), and the comparatively subtle peaks at 26.22 ppm and 21.53 ppm were referred to methine and methyl groups, respectively [27,28].

Regarding the resonance variations after the treatments, the first phenomenon observed was that all three treated samples demonstrated broader spectra than untreated WPC, which might be attributed to the less conformational exchange and rotational diffusion in the rigid phase [22]. As expected, when compared to the untreated counterpart, the peak intensity at 32.51 ppm in the spectrum of MAPE treated sample dramatically increased, indicating that MAPE was covalently bonded to wood particles. Apart from that, the more prominent signal at 172.47 ppm was not only contributed by the more resolved lignin units, but should be also resulted from the introduced MA groups in MAPE and ester linkages formed between the MA groups and hydroxyl groups of wood. These results were in a good agreement with the above FTIR analysis of MAPE treatment. The reaction mechanism of MAPE coupling agent with wood flour and PE could be explained as the activation of the copolymer by heating followed by the esterification of wood particles. This treatment increases the surface energy of wood flour to a level much closer to that of the matrix, and thus results in better wettability and enhances interfacial adhesion between the filler and matrix [29]. 

The incorporation of Si69 into the composites were discriminated by the appearance of resonance signals at 51.50 ppm, 49.18 ppm, and 47.23 ppm, which were attributed to the C–S_*x*_ bonds, including C–S and C–S–S existed in Si69 molecules, and the C–S_*x*_ bonds formed between the dissociated coupling agent and polymer chains in the matrix (Figure 2b) [30,31,32]. Compared to the spectrum of the untreated sample, the new shoulder at 60.50 ppm was probably contributed by Si–O–C bonds, which existed in Si69 and covalently formed between Si69 and wood flour [33,34]. These spectral characteristics unveiled the reactions between Si69 and two constituents of the composite, which were unfortunately not discerned from FTIR analysis probably due to overlapping of diagnostic signals and insufficient concentrations of these bonds to be detected. The proposed corresponding reactions were presented in Figure 2b. The ethoxy groups of Si69 first reacted with functional groups (mainly hydroxyl) of wood flour to form a siloxane bond, thus the sulfide group of Si69 bonded wood particle was dissociated and reacted with PE molecules to form a crosslink between wood and matrix. Si69 has a sulfidic linkage of di- to octa-sulfides and the average number of S_*x*_– is about 3.8. Polysulfides in Si69 could be dissociated at low temperature (near room temperature) to form radicals, and thus reacted with polymer molecules with storage time elapses. Therefore, the polysulfides of retained silane in the composite should be able to be dissociated and further react with both PE molecules and unreacted functional groups on wood surface [35]. 

In the spectrum of VTMS treated WPC, the peak at 133.80 ppm was initially assigned to lignin units in wood particles. In addition to the more resolved signal after this treatment, the strengthened intensity of this peak was also resulted from the incorporated carbons of Si–C=C and O–Si–C=C within VTMS structure [34,36]. The reaction between VTMS and wood flour (Figure 2c) was confirmed by the occurrence of the peaks at 68 ppm and 58 ppm, since which were attributed to the carbons of Si-O–C [33,34]. The considerable enhancement of the intensity at 32.51 ppm (–CH_2_–) suggested that the VTMS had successfully bonded to PE chains via its unsaturated C=C groups.

It should be pointed out that the relative resonance signals of crystalline carbohydrates (around 89 ppm and 65 ppm) to amorphous moieties (around 84 ppm and 63 ppm) decreased to some extent after the treatments, which might be an indication of the disordering of cellulose and the conversion to an amorphous state under these treatments. Transformation of crystalline cellulose to an amorphous state in hot and compressed water had been reported recently, which was determined as a consequence of the synergetic effect between the thermal properties of crystalline cellulose and the unique properties of hot and compressed water [37]. It was reported that in the NMR spectra of g-methacryloxypropyl trimethoxy silane (MPS) grafted cellulose, the spectral signals of the grafted MPS and amorphous cellulose were emphasised after this silane treatment, while the crystalline cellulose form drastically decreased [38]. The possible explanation of this diminishing of relative resonance intensity of crystalline carbohydrates was that VTMS may penetrate into wood lumens and vessels, thus reacted with the functional groups of cellulose, which in the meantime underwent transformation into an amorphous form under high temperature and pressure. 

### 3.2. Interface Bonding Scenarios and Mechanisms 

**Figure 5:**
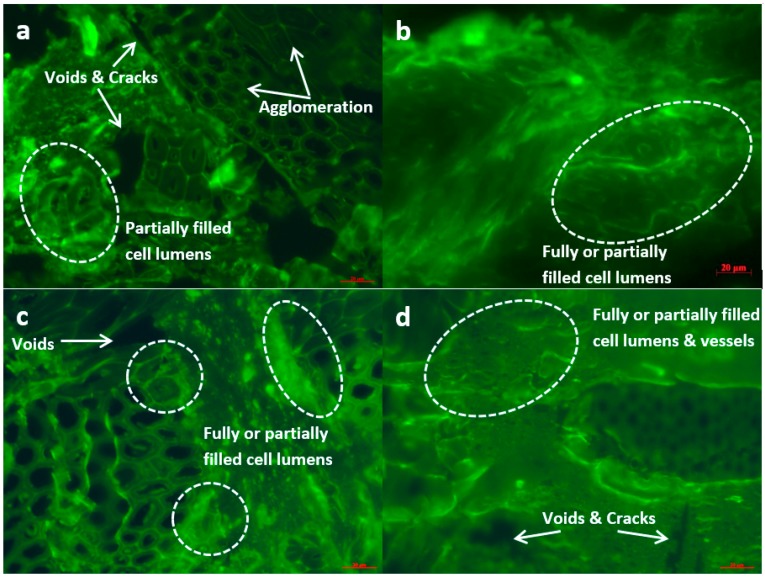
Fluorescence Microscope (FM) photographs of cross section of untreated (**a**), MAPE treated (**b**), Si69 treated (**c**), and VTMS treated (**d**) composites.

**Figure 6:**
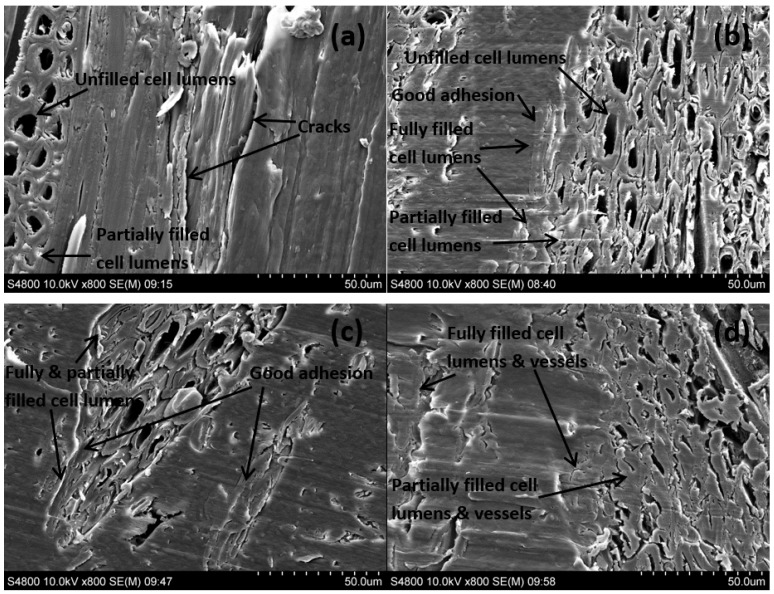
SEM photographs of cross section of untreated (**a**), MAPE treated (**b**), Si69 treated (**c**), and VTMS treated (**d**) composites.

The improvement of the chemical adhesion and compatibility between the constituents of WPC resulted from the chemical bonding and reactions between the incorporated coupling agents and the constituents of WPC could be anticipated to contribute to the interface refinery. The effect of the coupling agent treatment on the interface bonding scenarios of the composites was scrutinised by the use of FM and SEM (Figure 5 and Figure 6). It can be seen a number of clear cracks or boundaries and voids between wood particles and the matrix occurred in the untreated WPC, which indicated a poor compatibility between the untreated raw materials. It was also observed that there were agglomerated wood particles unevenly distributed in the matrix due to the readily formed hydrogen bonds within uncompatibilised wood particles [16]. In addition, although there were a few cell lumens that were partially filled by the polymer resin, the majorly unfilled cell lumens along with the existence of micro cracks between wood and PE denoted the improper interfacial adhesion of the untreated WPC. Comparatively, in the treated WPC (Figure 5b–d and Figure 6b–d), the wood flour was completely wetted by the matrix and firmly bonded to it, demonstrating superior interfacial adhesion with resin impregnation throughout the interface. More importantly, a large number of cell lumens of these samples were discerned to be partially or utterly filled by the resin, which again confirmed the enhanced interfacial adhesion and also the compatibility and wettability improvements. 

It was interesting to notice that there existed the deformed cell lumens in the treated WPC, especially the VTMS treated sample (Figure 6d), which should be resulted from the intensified pressure and compression of the interface regions during the treatments. Apart from the cell lumens, the vessels of the wood particles in VTMS treated sample were also completely or partially filled with PE resin. It was speculated that the coupling agent treatments would provide the resin with better fluidity due to the crosslinking between the hydrophobic part of coupling agent and the polymer chains of PE under high temperature and pressure, and the deformed lumens and vessels tended to facilitate the flow of resin in random directions. It has been reported that in the case of radial and tangential penetration of UF (urea-formaldehyde) adhesives into poplar wood, the resins preferably filled the wood vessels, rather than the wood fibres when the wood fibres and vessels close to the bond line were deformed [39,40]. 

Hydrodynamic flow of molten PE resin in the composites was initiated by an external compression force through vessels, and then proceeded into the interconnected network of cell lumens and pits in interface region, with flow moving primarily in the paths of least resistance [39,40]. The flow paths in any directions were in general a combination of open cut lumens and vessels, as well as of large pits.

**Figure 7:**
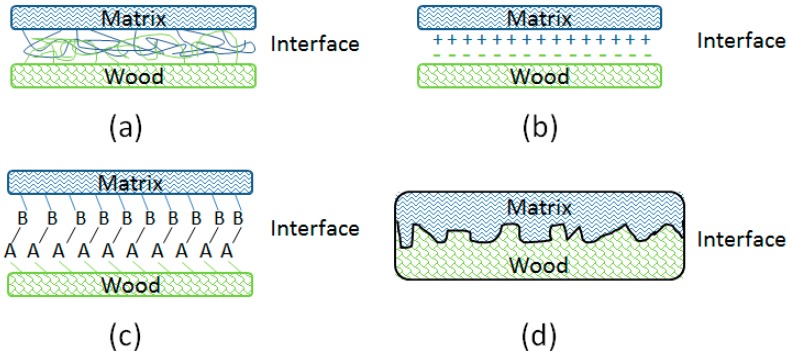
Wood-matrix interfacial bonding mechanisms: (**a**) molecular entanglement following interdiffusion, (**b**) electrostatic adhesion, (**c**) chemical bonding, and (**d**) mechanical interlocking.

The interface region formed between wood flour and matrix is in fact a zone of compositional, structural, and property gradients, in which a set of processes occur on the atomic, microscopic, and macroscopic levels. It has been recognised to play a predominant role in governing the global composite behaviour by controlling the stress transfer between wood and matrix, and is primarily dependent on the level of interfacial adhesion. The wood-matrix interfacial bonding mechanisms were assumed to include interdiffusion, electrostatic adhesion, chemical reactions, and mechanical interlocking (Figure 7), which together were responsible for the interfacial adhesion.

Interdiffusion was developed on the basis of good wetting of wood particle (Figure 6) through intimate intermolecular interactions between the molecules of wood and polymer, e.g., hydrogen and covalent bonding, electrostatic, and Van der Waals forces. Electrostatic adhesion was attributed to the creation of opposite charges (anionic and cationic) on the interacting surfaces of wood and polymer matrix; thus, an interface consisting of two layers of opposite charges was formed, which accounted for the adhesion of two constituents of the composite. Chemisorption occurred when chemical bonds, including atomic and ionic bonds, such as C–O–C, C–S, and Si–O–C covalent bonds, were created between the substances of the composite as a result of chemical reactions. Mechanical interlocking took place through the resin penetration into the peaks, holes, valleys, and crevices or other irregularities of the substrate, which can be seen in the FM and SEM images (Figure 5 and Figure 6), then anchored itself through solidification.

The distinguished enhancement of interfacial bonding of maleated and silane coupling agents treated WPC could be explained as follows: the hydrophilic moiety in the coupling agents reacted with the functional groups of wood flour to form covalent linkages, while the hydrocarbon chains crosslinked with the polymer matrix to create molecular entanglements. Specifically, as thoroughly discussed in the above section of chemical structure and bonding, the MA moiety in MAPE, the ethoxy groups of Si69 and the methoxyl groups of VTMS reacted with the hydroxyl groups of wood flour, in the meantime, the grafted PE chains in MAPE, the dissociated sulfide groups in Si69, and the vinyl groups of VTMS chemically bonded and/or interacted with the PE macromolecules. Thus, the extent and degree of interdiffusion between wood and PE molecules were increased due to the better chemical compatibility resulted from these chemical reactions and the more flexibility of interchains explored in the NMR analysis (Section 3.1.2). The introduced hydrocarbon chains of coupling agents also led to the decrease of hydrophilicity and the increase of surface energy of wood flour, and improved the chemical affinity of the matrix, thereby resulted in enhanced wettability of the wood by the resin and interfacial adhesion [29,41,42]. In addition, more contact areas between wood and matrix were created for resin penetration and mechanical interlocking of the substrate (Figure 5 and Figure 6). The more deformed cell lumens and vessels in VTMS treated sample should result in stronger mechanical interlocking than MAPE and Si69 treated samples, due to the increased resin penetration. In fact, an increase in any bonding mechanism (i.e., interdiffusion, chemical reactions, mechanical interlocking, etc.) would inevitably give rise in the enhancement of other bonding systems/mechanisms, which mutually accounted for the interfacial bonding refinery. 

The influence of the coupling agent treatments on the mechanical properties and the correlation between the interface structure and performance of the composites have been comprehensively studied in our previous work [43]. In short, the enhanced interfacial bonding due to the coupling agent treatments resulted in improvements of bulk mechanical properties, such as tensile strength, tensile modulus, and storage modulus, while the in situ mechanical properties of the composites were subject to a number of phenomena including fibre weakening or softening impact, crystalline structure transformation and cell wall deformation. 

## 4. Conclusions

The interfaces of WPC were optimised by the incorporation of MAPE, Si69 and VTMS coupling agents. FTIR and NMR results confirmed the chemical reactions between the coupling agents and the constituents of the composites, i.e., covalent bonding with the functional groups (mainly hydroxyl groups) of wood flour and crosslinking with PE molecules. The crosslinking between the coupling agents and PE matrix under high temperature and pressure might give rise to better fluidity of the resin, and thus facilitate its hydrodynamic flow in interface region. The treated composites possessed better interfacial adhesion by showing completely polymer coated wood flour, resin impregnation throughout the interface, and the partially and fully resin filled cell lumens. The enhanced interface of the composites after the coupling agent treatments was resulted from the combination of improved interdiffusion, electrostatic adhesion, chemical reactions, and mechanical interlocking. 

## Figures and Tables

**Table 1:** Formulation of the composites.

Sample	Wood (%)	PE (%)	TPW 709 (%)	12HSA (%)	MAPE (%)	Si69 (%)	VTMS (%)
Untreated WPC	50	43	3.5	3.5	0	0	0
MAPE treated WPC	50	40	3.5	3.5	3	0	0
Si69 treated WPC	50	40	3.5	3.5	0	3	0
VTMS treated WPC	50	40	3.5	3.5	0	0	3
